# Tolerance to Ammonia of *Thulinius ruffoi* (Bertolani, 1981), a Tardigrade Isolated from a Sewage Treatment Plant

**DOI:** 10.1007/s00128-015-1593-6

**Published:** 2015-07-09

**Authors:** Mateusz Sobczyk, Klaudia Michno, Paulina Kosztyła, Daniel Stec, Łukasz Michalczyk

**Affiliations:** Institute of Environmental Sciences, Jagiellonian University, Gronostajowa 7, 30-387 Kraków, Poland; Department of Entomology, Institute of Zoology, Jagiellonian University, Gronostajowa 9, 30-387 Kraków, Poland

**Keywords:** Acute toxicity, LC_50_, NH_3_, NH_4_^+^, Tardigrada

## Abstract

The acute toxicity of ammonia on *Thulinius ruffoi* (Bertolani, 1981), a eutardigrade isolated from a small waste water treatment plant (WWTP) in Poland, was estimated. Our results show that no active individuals survived a 24 h exposure to solutions equal to or higher than 125 mg/L of total ammonia nitrogen (NH_3_–N + NH_4_^+^–N), which, under the conditions in our experiment, was equivalent to 1.17 mg/L of un-ionised ammonia (NH_3_). The LC_50_ concentration of total ammonia nitrogen was equal to 52 mg/L (or 0.65 mg/L un-ionised ammonia). Given that the norms for the concentration of ammonia in treated waters leaving WWTPs are usually several times lower than the LC_50_ for *T. ruffoi*, this species does not seem to be a good bioindicator candidate for WWTPs. In this paper we also note that various ecotoxicological studies use different methodological approaches and we suggest that a more uniform methodology may aid interspecific comparisons of LC_50_ values.

Tardigrada are a phylum of microscopic, eight-legged invertebrates, typically ca. 0.5 mm in length. Currently, there are over 1200 described species of these metazoans, classified with other moulting animals in the megaclade Ecdysozoa (Degma et al. 2014; Aguinaldo et al. 1997). Commonly called water bears, they are distributed globally and can live in an overwhelming majority of habitats (Michalczyk 2012; Nelson et al. 2015). They are found in marine and brackish waters as well as in freshwater and terrestrial habitats in a wide range of moisture regimes (Nelson and Marley 2000). The vast majority of described tardigrade species are terrestrial and live in at least periodically moist habitats such as soil, leaf litter, mosses and lichens. Although tardigrades are a poorly investigated phylum, they are widely known for their ability to enter cryptobiosis, a latent state in which virtually no metabolic activity can be detected (reviewed in Wełnicz et al. 2011). Nevertheless, despite their cryptobiotic abilities, tardigrades require liquid water in order to be active.

Tardigrada, along with Rotifera, Nematoda, Gastrotricha and Annelida, are an example of metazoans that may also inhabit sewage treatment plants, specifically activated sludge (i.e. particles and flocs formed in wastewater by microorganisms growing on inorganic and organic particles in aeration tanks) (Nelson et al. 2015). Of all these phyla, water bears have been recorded least frequently, e.g. Chen et al. (2004) found tardigrades only in 4 out of 200 samples (2 %) taken from five municipal sewage treatment plants in Beijing during 1 year, whereas rotifers, nematodes, gastrotrichs and annelids occurred, respectively, in 83 %, 21 %, 20 % and 9 % of collected samples. Nematoda, Rotifera and Annelida probably play an important role as predators of bacterial cells (Poole 1984; Ratsak et al. 1993), but their significance in the purification process is still not well understood compared to that of protozoa (e.g. Pauli et al. 2001). The role of tardigrades in the activated sludge process is also unclear because of the poor knowledge of their biology and very low occurrence rates in aeration tanks. According to Utsugi (2001), the tardigrade *Isohypsibius myrops* (Du Bois-Reymond Marcus, 1944), observed in Japanese treatment plants, seemed to live and reproduce in sewage aeration tanks as a scavenger. Most of what is known about tardigrades in activated sludge is based on a manual for activated sludge microscopic investigations by Jenkins et al. (2003). The manual states that: (1) tardigrades, probably due to their susceptibility to ammonia toxicity, occur only in nitrifying activated sludge systems, and that (2) overabundance of tardigrades (along with nematodes, gastrotrichs and annelids) is an indicator of a low food-to-microorganism ratio (the ratio of food entering the activated sludge process to the abundance of unicellular organisms in aeration tanks; Gerardi 2003) and of a high mean cell residence time (the amount of time that solids or bacteria are maintained in the activated sludge process; this measure is also known as the solids retention time; Gerardi 2003). Tardigrades were occasionally observed at low sludge loading levels, i.e. below 0.1 kg BOD/kg MLSS/day (where BOD stands for biological oxygen demand and MLSS for mixed liquor suspended solids; Eikelboom 2000). In terms of tardigrade diversity, the number of species found in wastewater plants is relatively low—so far only two species have been recorded in this type of habitat: “*Macrobiotus* sp.” (in several US treatment plants; Jenkins et al. 2003) and *Isohypsibius myrops* (Du Bois-Reymond Marcus, 1944), earlier incorrectly determined as *Macrobiotus* sp., in several treatment plants in Tokyo and other areas of Japan (Utsugi 2001). Given that *Macrobiotus* species are typically terrestrial, what Jenkins et al. (2003) probably found was a misidentified species of *Dactylobiotus*, which is an exclusively freshwater genus that was separated from the genus *Macrobiotus* by Schuster et al. (1980).

Most of the nitrogen in municipal and many industrial wastewaters occurs in the form of ionised ammonia (NH_4_^+^), which is in a pH and temperature dependent equilibrium with un-ionised ammonia (NH_3_) or is bound with urea or other organic molecules such as amino acids (Seviour and Nielsen 2010). The toxicity of the solution correlates positively with the amount of un-ionised ammonia (NH_3_), which increases with pH and temperature (Hellawell 1989). Ionised ammonia is only moderately poisonous to aquatic animals (Abel 2002), whereas un-ionised ammonia is very toxic and its negative effects are widely described in literature (Vedel et al. 1998; Marttinen et al. 2002; Arauzo and Valladolid 2003). Ammonia is a common cause of fish death but also low concentrations of ammonia are also known to negatively affect fish growth, gill condition and haematocrit parameters (Milne et al. 2000). The value of total ammonia measured analytically in water and wastewater is in fact the sum of both the un-ionised NH_3_ and the ionised NH_4_^+^, i.e. so called “total ammonia” (NH_3_ + NH_4_^+^).

Given that organisms inhabiting aeration tanks in sewage treatment plants are used as bioindicators and as means of improving the treatment process, numerous ecotoxicological studies investigating ammonia effects on protozoans and rotifers as well as on some arthropods have been published (e.g. Snell and Janssen 1995; Xu et al. 2004; Xiang et al. 2010). However, in contrast to the metazoan phyla mentioned above, no data on ammonia effects on tardigrades are available and the aim of the present study is to fill this gap. Specifically, under controlled laboratory conditions we estimated the mean concentration of ammonia over a 24 h exposure, resulting in a 50 % mortality (LC_50_) of adult individuals of *Thulinius ruffoi* (Bertolani, 1981), a tardigrade isolated from a sewage treatment plant in southeastern Poland. This paper presents not only the first assessment of ammonia toxicity in a species of Tardigrada, but it is also the first ever fully controlled ecotoxicological study performed on active tardigrades. In this paper we also strongly encourage researchers to use more cohesive methodology in order to facilitate comparisons between different studies addressing ammonia toxicity.

## Materials and Methods

*Thulinius ruffoi* (Bertolani, 1981), originally described as *T. ruffoi* from the Sesia River in northwestern Italy, has been reported from several sites in Italy, Greece, Poland, Sweden, northwestern Russia and the eastern USA (Kaczmarek et al. 2010). *T. ruffoi* was first recorded in Poland from decaying leaves in a post-meteor freshwater pond in the “Meteoryt Morasko” Nature Reserve in western Poland (Kaczmarek and Michalczyk 2005). Thus, this is the second record of this species in Poland and the first ever report of *T. ruffoi* from a wastewater plant. The species was identified with the use of the diagnostic key in Kaczmarek et al. (2010) and the original description by Bertolani (1981). *T. ruffoi* is an exclusively aquatic species and it is not capable of cryptobiosis (e.g. anhydro- or cryobiosis), although under unfavourable conditions the animals may form cysts, however these latent forms are not truly cryptobiotic (Pigoń and Węglarska 1955).

Specimens for the present study were isolated from activated sludge collected from a small wastewater treatment plant in Zelków, near Kraków (southeastern Poland, 50°09′52′′N, 19°48′26′′E). After isolation under a stereomicroscope, tardigrades were washed several times with ddH_2_O and transferred to 5 cm Ø plastic Petri dishes (ca. 100 animals/dish) with a mixture of distilled and spring water “Żywiec Zdrój” (3:1) and unicellular freshwater algae *Chlorococcum* sp. and *Chlorella* sp. (1:1, Sciento, UK) provided ad libitum. In order to facilitate tardigrade locomotion, Petri dish bottoms were scratched with fine sandpaper. The Petri dishes were placed in a *TK 600* *Nüve* climatic chamber at 16°C (i.e. average yearly temperature in aeration tanks of the Zelków treatment plant). Water and food were changed fortnightly and tardigrade density was kept at ca. 100 individuals per dish. In order to allow accommodation to lab conditions prior to the experiment, tardigrades were cultured for 4 months, which is equivalent to ca. six overlapping generations. The pilot study, in which we observed the entire life cycle of 24 isolated individuals, showed that all animals were parthenogenetic females.

In order to investigate ammonia toxicity to *T. ruffoi*, we prepared a series of nine ammonium test solutions. First, crystallised NH_4_Cl (*POCH, Poland*) was placed in an open container at 110°C for 4 h in order to minimise the amount of water bound in crystals. Next, 1.909 g of ultra-dry NH_4_Cl was dissolved in 1 L of ddH_2_O to obtain a 1000 NH_3_–N + NH_4_^+^–N (total ammonia nitrogen) mg/L stock solution. Test solutions were prepared by pouring appropriate volumes of the stock into clean glass graduated cylinders and then adding ddH_2_O up to a 100 mL and mixing the solution in a clean beaker. The solutions were as follows: 0 (control), 25, 50, 75, 100, 125, 150, 175, and 200 NH_3_–N + NH_4_^+^–N mg/L. These solutions are equivalent to 0.00, 0.18, 0.36, 0.54, 0.73, 0.91, 1.09, 1.27, 1.45 NH_3_–N (un-ionised ammonia nitrogen) mg/L, respectively (Emerson et al. 1975; Xiang et al. 2010 after Albert 1973). Given that adding NH_4_Cl to water decreases the pH of the solution, all solutions (including control) were equalised to pH 7.4 (i.e. same as the pH in the original aeration tank) by adding minute amounts of NaOH and continuous monitoring of the pH with a *handylab pH 11* pH-meter (*Si Analytics GmbH*). Then, the concentration of total ammonia was estimated with a *Slandi LF205* spectrophotometer and an *Ammonium Cell Test* (*A6/25, WTW GmbH, Germany*) immediately before and after the survival assay. Both spectrophotometer readings were almost identical (r^2^ = 0.99) and they were also equally highly correlated with the nominal concentrations (r^2^ = 0.99).

Given that in ecotoxicological literature four different measures of ammonia concentrations are used, we provide all of them in order to aid comparisons of our results with future studies. These measures are as follows:

1. Total ammonia nitrogen-N (NH_3_–N + NH_4_^+^–N), raw data, according to the nominal scale;
2. Total ammonia (NH_3_ + NH_4_^+^), calculated by dividing the total ammonia nitrogen concentrations (no. 1) by the value of 1.29 (APHA 2005);
3. Un-ionised ammonia nitrogen-N (NH_3_–N), calculated using the general equation of bases (Xiang et al. 2010 after Albert 1973):
  $${\text{NH}}_{3} { = }\frac{{\left[ {{\text{NH}}_{3} + {\text{NH}}_{4}^{ + } } \right]}}{{\left[ {1 + 10^{{pKa - {\text{pH}}}} } \right]}},$$
  with the value in the numerator being the concentration of the total ammonia nitrogen (no. 1) and the *pKa* term calculated according to Emerson et al. (1975): *pKa* = 0.09018 + 2729.29/T, where temperature (T) is given in Kelvins;
4. Un-ionised ammonia (NH_3_), calculated using the general equation of bases same as in no. 3 but with the value in the numerator being the concentration of the total ammonia (no. 2) and the *pKa* term calculated according to equation provided in no. 3.

Mature (ca. 3–5 instar) and well-fed (i.e. with intestine filled with algae) animals of similar size were isolated from the stock culture, washed in ddH_2_O and randomly assigned to the nine experimental treatments (including control). In each treatment there were ten Eppendorf test vials, each with 1.5 mL of a given NH_4_Cl solution. To each vial five tardigrades were transferred in 20 µL of ddH_2_O, using a semi-automatic pipette. Thus, in each treatment there were 50 tardigrades (10 replicates × 5 tardigrades), which amounted to 450 tardigrades in the entire experiment (50 tardigrades × 9 treatments). After 24 h, individuals were transferred to Petri dishes and examined under a *Nikon SMZ800* stereomicroscope with the dark field illumination (magnification 60×). Animals were classified as alive (if they were moving) or dead (when no motion could be detected and animals were completely stretched out as in anoxybiosis or when dead). Given that tardigrades devoid of oxygen are also turgid and motionless, we transferred all animals classified as dead to 24-well plastic plates filled with fresh aerated ddH_2_O. Animals were checked again after another 24 h, however none of them showed any signs of life and tissue lysis at this stage was already clearly visible. This confirmed that the original classification of dead and live animals was correct. The entire survival assay was conducted at 16°C.

The LC_50_ values were computed in R (version 3.0.2, R Development Core Team 2008), using the MASS library with the *dose.p* function and by fitting our data to a probit model (Venables and Ripley 2002). The 95 % CIs were calculated according to Łomnicki (2002).

The most straightforward way of comparing our results to those from the literature would be to use a single common ammonia concentration measure. Unfortunately, however, different studies have provided their results in different units. Whereas the transformation of total ammonia nitrogen (NH_3_–N + NH_4_^+^–N) to and from total ammonia (NH_3_ + NH_4_^+^) is a matter of simple proportion, the calculation of either un-ionised ammonia nitrogen (NH_3_–N) or un-ionised ammonia (NH_3_) to and from total ammonia nitrogen (NH_3_–N + NH_4_^+^–N) requires pH and temperature readings, which are not provided in the majority of studies. Thus, we were unable to use a single measure of ammonia concentration and, depending on units provided in a given paper, we transformed literature data either into total ammonia nitrogen (NH_3_–N + NH_4_^+^–N) or into un-ionised ammonia (NH_3_) and compared our results separately for each of the two measures.

## Results and Discussion

All tardigrades survived in the control treatment, whereas those exposed to increasing concentrations of ammonia showed decreasing survival, with all individuals dead at a concentration of 125 mg/L NH_3_–N + NH_4_^+^–N and higher (which is equivalent to 161.25 mg/L NH_3_ + NH_4_^+^, 0.91 mg/L NH_3_–N, and 1.17 mg/L NH_3_). Thus, in order to calculate LC_50_, only concentrations from 0 to 125 mg/L were included in the analysis. Estimates of ammonia LC_50_ for *T. ruffoi* expressed in four measures of ammonia concentration are provided in Table 1.

**Table 1:** Means ± 95 % CIs for estimates of ammonia LC_50_ for *Thulinius ruffoi* (Bertolani, 1981) expressed in four measures of ammonia concentration (at pH 7.4 and temperature 16°C)

Measure of nitrogen concentration in the solution	Lower 95 % CI (mg/L)	Mean LC_50_ (mg/L)	Upper 95 % CI (mg/L)
Total ammonia nitrogen (NH_3_–N + NH_4_^+^–N)	46.66	51.98	57.89
Total ammonia (NH_3_ + NH_4_^+^)	60.22	67.08	74.71
Un-ionised ammonia nitrogen (NH_3_–N)	0.45	0.51	0.56
Un-ionised ammonia (NH_3_)	0.59	0.65	0.73

The majority of studies investigating the toxic effects of ammonia on organisms living in wastewater treatment plants (WWTPs), conducted by the staff of WWTPs, use total ammonia nitrogen (NH_3_–N + NH_4_^+^–N) as a measure of ammonia concentration, without paying much attention to un-ionised ammonia (NH_3_) (Puigagut et al. 2005, 2009; Klimek et al. 2012). This is somewhat surprising as it is actually NH_3_ that is toxic to life whereas the effects of total ammonia are much less profound (Hellawell 1989). In contrast, the majority of laboratory experiments use the concentration of un-ionised ammonia (NH_3_) in order to estimate the toxic effects of ammonia. Unfortunately, however, both WWTP and lab studies rarely provide temperature and pH of the solutions, thus making it impossible to convert one measure to the other and to use a single universal toxicity scale. Therefore, we are forced to compare and discuss the results of our experiment separately with studies reporting LC_50_ values for the total ammonia nitrogen and un-ionised ammonia.

We were able to find only six studies that investigated the effects of total ammonia nitrogen on protozoans and metazoans (Table 2). With the LC_50_ of ca. 52 mg/L NH_3_ + NH_4_^+^ (Table 1), *T. ruffoi* is similar in its tolerance to total ammonia to three crawling and one attached ciliate species and to grey mullet, a species of a ray-finned fish (Table 2). The LC_50_ values for these species, including *T. ruffoi*, vary from 20 to 80 mg/L NH_3_–N + NH_4_^+^–N, with an average of 46 mg/L NH_3_–N + NH_4_^+^–N. Of all tested taxa, only the swimming ciliate *Coleps hirtus*, with an LC_50_ of 344 mg/L NH_3_–N + NH_4_^+^–N, stands out in its resistance to total ammonia.

**Table 2:** A comparison of the sensitivity of various species to total ammonia nitrogen (NH_3_–N + NH_4_^+^–N) from the literature with data for *Thulinius ruffoi* obtained in the present study (in bold)

Species (phylum)	LC_50_ concentration (NH_3_–N + NH_4_^+^–N mg/L)	Exposure time (h)	Source
*Chilodonella uncinata* (Ciliophora)	20	48	Puigagut et al. (2005)
*Stentor coeruleus* (Ciliophora)	33	24	Klimek et al. (2012)
*Mugil platanus* (Chordata)	41	24	Sampaio et al. (2002)
*Acineria uncinata* (Ciliophora)	50	48	Puigagut et al. (2005)
***Thulinius ruffoi*** **(Tardigrada)**	**52**	**24**	**Present study**
*Aspidisca costata* (Ciliophora)	80	48	Puigagut et al. (2005)
*Coleps hirtus* (Ciliophora)	344	24	Klimek et al. (2012)

Taxa are listed from most to least sensitive to ammonia. Please note that the LC_50_ for three of five ciliate species was measured after a 48 h exposure, thus their LC_50_ values would be probably higher for a 24 h exposure

European Water Framework Directive norms require that total nitrogen in treated water leaving WWTPs cannot exceed 15 mg/L for small treatment plants (10,000–100,000 population equivalent) and 10 mg/L for large WWTP (over 100,000 population equivalent) (The Urban Waste-water Treatment Directive 91/271/EEC). Thus, *T. ruffoi*, with its LC_50_ value approximately five times higher than allowed levels for large WWTPs, does not seem to be a promising candidate as a bioindicator of total ammonia concentration in the activated sludge process. Nevertheless, the effects of longer exposure to total ammonia (e.g. over the entire life cycle) are needed in order to fully assess the utility of *T. ruffoi* as a bioindicator in WWTPs, because it is very likely that concentrations much lower than the LC_50_ estimated in this study for a 24 h exposure may cause infertility or even mortality if animals are exposed to ammonia for longer periods of time.

Compared to the total ammonia, there are twice as many studies on the toxic effects of un-ionised ammonia (Table 3). What is more, these studies were performed using a wider range of taxa, comprised of five arthropod, three chordate, two rotifer and one ciliate species. Species listed in Table 3 exhibit a very wide range of tolerance to un-ionised ammonia, with no apparent pattern relating to their phylogenetic position. They can be grouped into three clusters of weakly, moderately and strongly resistant to un-ionised ammonia. The majority of tested species, including *T. ruffoi*, fall into the first group, with LC_50_ values ranging from to 0.28 to 3.21 mg/L NH_3_ and the mean for the group being 1.41 mg/L NH_3_. The moderately resistant group consists of only two species, a mayfly and a marine ciliate (range 8.20–10.09, mean 9.15 mg/L NH_3_). Finally, the cluster of the most resistant organisms consist of two species, a euryhaline rotifer and a hyporheic copepod (range 17.70–18.63, mean 18.17 mg/L NH_3_). It is worth noting that the already high LC_50_ for the copepod *Bryocamptus zschokkei* was measured over 96 h, thus the LC_50_ for a period of 24 h would be probably even higher. Thus, overall, *T. ruffoi*, with LC_50_ of ca. 0.65 mg/L NH_3_, can be classified as very sensitive to un-ionised ammonia.

**Table 3:** A comparison of the sensitivity of various species to un-ionised ammonia (NH_3_) from the literature with data for *Thulinius ruffoi* obtained in the present study (in bold)

Species (phylum)	LC_50_ concentration (NH_3_ mg/L)	Exposure time (h)	Source
*Salmo saltar* (Chordata)	0.28	24	Herbert and Shurben (1963)
***Thulinius ruffoi*** **(Tardigrada)**	**0.65**	**24**	**Present study**
*Salmo gairdeneri* (Chordata)	0.70	24	Herbert and Shurben (1963)
*Mugil platanus* (Chordata)	0.95	24	Sampaio et al. (2002)
*Eulimnogammarus toletanus* (Arthropoda)	1.23	24	Alonso and Camargo (2004)
*Daphnia similoides* (Arthropoda)	1.59	24	Xiang et al. (2010)
*Gammmarus pulex* (Arthropoda)	2.64	24	Williams et al. (1986)
*Brachionus calyciflorus* (Rotifera)	3.21	24	Snell and Janssen (1995)
*Baetis rhodani* (Arthropoda)	8.20	24	Khatami et al. (1998)
*Euplotes vannus* (Ciliophora)	10.09	24	Xu et al. (2004)
*Brachionus plicatilis* (Rotifera)	17.70	24	Snell and Janssen (1995)
*Bryocamptus zschokkei* (Arthropoda)	18.63	96	Di Marzio et al. (2009)

Taxa are listed from most to least sensitive to ammonia. Please note that the LC_50_ for the most resistant species listed was measured after a 96 h exposure to ammonia, thus its LC_50_ value would be probably higher for a 24 h exposure

The review of the ecotoxicological literature investigating the effects of ammonia on living organisms shows the lack of a cohesive approach to the subject. Some studies use a measure of total ammonia whereas other use a measure of un-ionised ammonia to calculate LC_50_ values. While the choice of unit of measurement is not important, the lack of associated data on temperature and pH of test solutions makes it impossible to convert one measure of ammonia concentration to another. An additional problem is that although the majority of studies use a 24 h period to calculate LC_50_ values, exposure times range from 24 to 96 h, which prevents direct comparisons between assays. Without a standardised methodology, a comparison of the results from different studies is virtually impossible. Therefore we would like to encourage researchers to (a) report temperature and pH of test solutions along with the units of ammonia concentrations and (b) assess mortality after a 24 h exposure, even if the experiment is designed to be conducted over a longer period of time.

Finally, while underlining that our study is the first to investigate the effect of ammonia (total and un-ionised) on the survival of tardigrades, we would like to emphasise the need for more studies on other aquatic tardigrade species as well. Ideally, such studies should not only try to assess LC_50_ values, but also look at the effects of ammonia on life history traits as these should give a greater insight into the role of ammonia in aquatic habitats.
